# Supplementary material for: Cross-presentation of dead cell-associated antigens shapes the neoantigenic landscape of tumor immunity
Source: Nat Immunol. 2026 Jan 2;27(1):72–81. doi: 10.1038/s41590-025-02354-w (PMC12764433; doi:10.1038/s41590-025-02354-w)
Supplement: Supplementary file 1 — Supplementary Table 1. [file 41590_2025_2354_MOESM1_ESM.pdf]

nature immunology

Supplementary information

https://doi.org/10.1038/s41590-025-02354-w

Cross-presentation of dead cell-associated antigens shapes the neoantigenic landscape of tumor immunity

In the format provided by the authors and unedited

Supplementary Table 1. Synthetic gBlock sequences for OVA fusion constructs used in antigen presentation studies.

OVA fusion constructs	gBlocks
**LA-OVA**	accccaagctggcctctgaggccgccaccatgggtgtcgctgatttgatcaagaagttcgagagcatcagcaaggaggaggcggccgccatgtccactcggacccagatcaacaaggtggtgag<br>attcgacaagctgcctggcttcggtgattccatcgaagcccaatgcggcacctcagtgaacgtgcacagtagcctgagggacatcctgaaccagatcacgaagcccaacgacgtgtactccttcag<br>tctggccagtcggctgtatgccgaagaacggtaccctatcctgcctgagtacctccagtgcgtgaaggagctgtaccgagggggtctggagcccatcaacttccagaccgccgcagatcaggctag<br>agaactgatcaacagctgggtggagtcccagaccaacggaatcatcaggaacgtgctgcagccttccagtgtggacagtcagactgccatggtgcttgtgaacgccatcgtcttcaagggcctgtgg<br>gagaaggcattcaaggacgaggatacccaggcaatgcccttccgggtgacagagcaggagagcaagcccgtgcagatgatgtaccagatcggactgttccgggtggcctccatggcatccgaa<br>aagatgaagatcctggagctgcccttcgcctcgggcacaatgtccatgctggtgctgctccctgacgaggtcagtggactggaacagctggagtcgatcatcaacttcgagaagctgaccgagtgga<br>cctcctccaacgtgatggaggagcgcaagatcaaggtgtacctgccgcggatgaagatggaggagaagtacaacctgacctccgtgctgatggccatgggcatcaccgatgtgttctcctcatccg<br>ccaacctgtcaggcatctcttccgctgagtccctgaaaatctcccaggccgtccacgccgctcatgctgagatcaacgaggccggtcgtgaagtagtggggtctgctgaggctggcgtggatgccgc<br>aagtgtgtctgaggaatttcgggccgaccaccccttcctcttctgcatcaagcacatcgccaccaatgccgtgctgttcttcggacgttgtgtgtctcctggcggaggaggctcccaccatcatcaccac<br>caccatcactaatgaggcctgtcaggccaagcttgggg
**mutLA-OVA**	accccaagctggcctctgaggccgccaccatgggtgtcgctgataaaatcaagaaggcggagagcgccagcaaggaggaggcggccgccatgtccactcggacccagatcaacaaggtggt<br>gagattcgacaagctgcctggcttcggtgattccatcgaagcccaatgcggcacctcagtgaacgtgcacagtagcctgagggacatcctgaaccagatcacgaagcccaacgacgtgtactcctt<br>cagtctggccagtcggctgtatgccgaagaacggtaccctatcctgcctgagtacctccagtgcgtgaaggagctgtaccgagggggtctggagcccatcaacttccagaccgccgcagatcagg<br>ctagagaactgatcaacagctgggtggagtcccagaccaacggaatcatcaggaacgtgctgcagccttccagtgtggacagtcagactgccatggtgcttgtgaacgccatcgtcttcaagggcct<br>gtgggagaaggcattcaaggacgaggatacccaggcaatgcccttccgggtgacagagcaggagagcaagcccgtgcagatgatgtaccagatcggactgttccgggtggcctccatggcatcc<br>gaaaagatgaagatcctggagctgcccttcgcctcgggcacaatgtccatgctggtgctgctccctgacgaggtcagtggactggaacagctggagtcgatcatcaacttcgagaagctgaccgagt<br>ggacctcctccaacgtgatggaggagcgcaagatcaaggtgtacctgccgcggatgaagatggaggagaagtacaacctgacctccgtgctgatggccatgggcatcaccgatgtgttctcctcat<br>ccgccaacctgtcaggcatctcttccgctgagtccctgaaaatctcccaggccgtccacgccgctcatgctgagatcaacgaggccggtcgtgaagtagtggggtctgctgaggctggcgtggatgc<br>cgcaagtgtgtctgaggaatttcgggccgaccaccccttcctcttctgcatcaagcacatcgccaccaatgccgtgctgttcttcggacgttgtgtgtctcctggcggaggaggctcccaccatcatcac<br>caccaccatcactaatgatgaggcctgtcaggccaagcttgggg
**Aff-OVA**	accccaagctggcctctgaggccgccaccatggcctctaactccctggaaatcgaggaactggccagattcgccgtggacgagcacaacaagaaagagaacgccctgctggaattcgtgcggg<br>tcgtgaaggccaaagaacagtcctctgtgccccactggtggtggaccaccatgtactatctgaccctggaagccaaggacggcggcaagaagaagctgtacgaggccaaagtgtgggtcaagcg<br>ggaccccaacatgatcttcaagattaacttcaaagagctgcaagagttcaagcccgtgggcgacgctgctggctctacaggatctagaggtggcggcggatccatgtctacccggacacagatcaa<br>caaggtcgtcagattcgacaagctgcccggcttcggcgattctatcgaggctcagtgtggcacctccgtgaacgtgcacagctccctgagagacatcctgaaccagatcaccaagcctaacgacgt<br>gtactccttcagcctggcctccagactgtacgccgaggaaagataccccatcctgcctgagtacctgcagtgcgtgaaagagctgtatagaggcggcctggaacctatcaacttccagaccgctgc<br>cgaccaggccagagagctgatcaattcctgggtcgagtcccagaccaacggcatcatcagaaacgtgctgcagccctccagcgtggactctcagacagctatggtgctggtcaacgccatcgtgtt<br>caaaggcctgtgggagaaagccttcaaggacgaggacacccaggccatgcctttcagagtgaccgagcaagagtctaagcctgtgcagatgatgtaccagatcggcctgtttagagtggcctccat<br>ggcctccgagaagatgaagatcctggaactgcctttcgcctccggcaccatgtctatgctggtgctgctgcctgatgaggtgtccggactggaacagctggaatccatcatcaacttcgagaagctga<br>ccgagtggacctcctccaacgtgatggaagaacggaagatcaaggtgtacctgcctcggatgaagatggaagagaagtacaacctgacctccgtgctgatggctatgggaattaccgacgtgttctc<br>cagctccgccaacctgtccggcatctcttctgccgagagcctgaagatctcccaggccgtgcatgctgctcacgccgagatcaatgaggccggcagagaagtcgtgggctctgctgaagcaggcg<br>tggacgctgcttctgtgtctgaggaattccgggccgaccatccttttctgttctgcatcaagcacattgccaccaacgccgtgctgttcttcggcagatgtgtgtctcctggcggaggcggctctcaccacc<br>atcatcaccatcaccactaatgaggcctgtcaggccaagcttgggg
**CrtAff-OVA**	accccaagctggcctctgaggccgccaccatggcctctaactccctggaaatcgaggaactggccagattcgccgtggacgagcacaacaagaaagagaacgccctgctggaattcgtgcggg<br>tcgtgaaggccaaagaacaggtggtggctggcaccatgtactatctgaccctggaagccaaggacggcggcaagaagaagctgtacgaggccaaagtgtgggtcaagccctgggagaacttca<br>aagagctgcaagagttcaagcccgtgggcgacgctggtggcggaggatctatgtctacccggacacagatcaacaaggtcgtcagattcgacaagctgcccggcttcggcgattctatcgaggctc<br>agtgtggcacctccgtgaacgtgcacagctccctgagagacatcctgaaccagatcaccaagcctaacgacgtgtactccttcagcctggcctccagactgtacgccgaggaaagataccccatc<br>ctgcctgagtacctgcagtgcgtgaaagagctgtatagaggcggcctggaacctatcaacttccagaccgctgccgaccaggccagagagctgatcaattcctgggtcgagtcccagaccaacgg<br>catcatcagaaacgtgctgcagccctccagcgtggactctcagacagctatggtgctggtcaacgccatcgtgttcaaaggcctgtgggagaaagccttcaaggacgaggacacccaggccatgc<br>ctttcagagtgaccgagcaagagtctaagcctgtgcagatgatgtaccagatcggcctgtttagagtggcctccatggcctccgagaagatgaagatcctggaactgcctttcgcctccggcaccatg<br>agtatgctggtgctgctgcctgatgaggtgtccggactggaacagctggaatccatcatcaacttcgagaagctgaccgagtggacctcctccaacgtgatggaagaacggaagatcaaggtgtacc<br>tgcctcggatgaagatggaagagaagtacaacctgacctccgtgctgatggctatgggaattaccgacgtgttctccagctccgccaacctgtccggcatctcttctgccgagagcctgaagatcagc<br>caggccgtgcatgctgctcacgccgagatcaatgaggccggcagagaagtcgtgggctctgctgaagcaggcgtggacgctgcttctgtgtctgaggaattccgggccgaccatccttttctgttctgc<br>atcaagcacattgccaccaacgccgtgctgttcttcggcagatgtgtgtctcctggcggcggaggctctcaccaccatcatcaccatcaccactaatgaggcctgtcaggccaagcttggg
