# Supplementary material for: Cross-presentation of dead cell-associated antigens shapes the neoantigenic landscape of tumor immunity
Source: Nat Immunol. 2026 Jan 2;27(1):72–81. doi: 10.1038/s41590-025-02354-w (PMC12764433; doi:10.1038/s41590-025-02354-w)
Supplement: Supplementary file 2 — Reporting Summary [file 41590_2025_2354_MOESM2_ESM.pdf]

nature portfolio

Corresponding author(s): Caetano Reis e Sousa

Last updated by author(s): 15/10/25

Reporting Summary

Nature Portfolio wishes to improve the reproducibility of the work that we publish. This form provides structure for consistency and transparency in reporting. For further information on Nature Portfolio policies, see our Editorial Policies and the Editorial Policy Checklist.

Statistics

For all statistical analyses, confirm that the following items are present in the figure legend, table legend, main text, or Methods section.

n/a	Confirmed	
☐	☒	The exact sample size (*n*) for each experimental group/condition, given as a discrete number and unit of measurement
☐	☒	A statement on whether measurements were taken from distinct samples or whether the same sample was measured repeatedly
☐	☒	The statistical test(s) used AND whether they are one- or two-sided<br>*Only common tests should be described solely by name; describe more complex techniques in the Methods section.*
☐	☒	A description of all covariates tested
☐	☒	A description of any assumptions or corrections, such as tests of normality and adjustment for multiple comparisons
☐	☒	A full description of the statistical parameters including central tendency (e.g. means) or other basic estimates (e.g. regression coefficient) AND variation (e.g. standard deviation) or associated estimates of uncertainty (e.g. confidence intervals)
☐	☒	For null hypothesis testing, the test statistic (e.g. *F*, *t*, *r*) with confidence intervals, effect sizes, degrees of freedom and *P* value noted<br>*Give P values as exact values whenever suitable.*
☒	☐	For Bayesian analysis, information on the choice of priors and Markov chain Monte Carlo settings
☒	☐	For hierarchical and complex designs, identification of the appropriate level for tests and full reporting of outcomes
☒	☐	Estimates of effect sizes (e.g. Cohen's *d*, Pearson's *r*), indicating how they were calculated

Our web collection on statistics for biologists contains articles on many of the points above.

Software and code

Policy information about availability of computer code

Data collection	n/a
Data analysis	The code used to analyse the data in this study is available in: https://github.com/FrancisCrickInstitute/DNGR1_XP

For manuscripts utilizing custom algorithms or software that are central to the research but not yet described in published literature, software must be made available to editors and reviewers. We strongly encourage code deposition in a community repository (e.g. GitHub). See the Nature Portfolio guidelines for submitting code & software for further information.

Data

Policy information about availability of data

All manuscripts must include a data availability statement. This statement should provide the following information, where applicable:

- Accession codes, unique identifiers, or web links for publicly available datasets
- A description of any restrictions on data availability
- For clinical datasets or third party data, please ensure that the statement adheres to our policy

Raw and processed data (whole exome sequencing) are submitted to European Nucleotide Archive (ENA) under the accession PRJEB100660.

Research involving human participants, their data, or biological material

Policy information about studies with human participants or human data. See also policy information about sex, gender (identity/presentation), and sexual orientation and race, ethnicity and racism.

Reporting on sex and gender	For the bioinformatic analysis of human cancer datasets, the association between CLEC9A and survival outcomes in patients with cancer were analysed using publicly available data downloaded from the cBioPortal for Cancer Genomics platform [http://cbioportal.org/], with source data from The Cancer Genome Atlas (TCGA) Pan-cancer Atlas. The focus of our analyses were on survival outcomes, including progression-free survival and overall survival, and were not segregated based on demographic features. All datasets include male and female patients.
Reporting on race, ethnicity, or other socially relevant groupings	See above.
Population characteristics	See above
Recruitment	n/a
Ethics oversight	n/a

Note that full information on the approval of the study protocol must also be provided in the manuscript.

Field-specific reporting

Please select the one below that is the best fit for your research. If you are not sure, read the appropriate sections before making your selection.

☒ Life sciences ☐ Behavioural & social sciences ☐ Ecological, evolutionary & environmental sciences

For a reference copy of the document with all sections, see nature.com/documents/nr-reporting-summary-flat.pdf

Life sciences study design

All studies must disclose on these points even when the disclosure is negative.

Sample size	We hypothesised that tumours developing in DNGR-1 KO mice were enriched in mutations in the n=88 genes encoding murine F-actin binding proteins (FABPs). Therefore, a power calculation for estimating the sample size required for MCA carcinogenesis model was performed by G.K. (Bioinformatics and Biostatistics team at the Francis Crick Institute), based on the following assumptions. There are in total 88 genes of interest encoding for FABPs. The Twist exome kit used for whole exome sequencing targets a total of 37,895,407bp or ~38Mb, with the 88 FABP genes comprising 334,760 of that, or 0.88% of the total region. Assuming that MCA fibrosarcomas have a high mutational burden of between 3,500 and 5,000 mutations based on previous literature, and the reference being aligned to is ~2.7Gb, a power calculation is generated using binomial probabilities: pbinom[0, N, prob=(burden*334760/2.7e9), lower=FALSE]. Therefore, it is estimated that n = 4–6 mice are required for each experimental arm, for in excess of 95% probability of at least one mouse having a variant within the FABP exon.<br><br>For experiments testing the tumour growth profile of individual primary cancer cell lines, at least n=3-5 mice were used per cohort, in line with previously published work, including by Schreiber and colleagues (Shankaran et al. 2001).<br>Likewise, for experiments testing the immunogenicity of neoantigen peptides in vivo or cross-priming of CD8 T cells in response to dead cell-associated antigen, at least n=3-5 mice were used per cohort.
Data exclusions	No data were excluded from the analyses.
Replication	All attempts at replication were successful, and where indicated, were noted in the figure legends.
Randomization	n/a
Blinding	n/a

Reporting for specific materials, systems and methods

We require information from authors about some types of materials, experimental systems and methods used in many studies. Here, indicate whether each material, system or method listed is relevant to your study. If you are not sure if a list item applies to your research, read the appropriate section before selecting a response.

Materials & experimental systems

n/a	Involved in the study	
☒	☐	Antibodies
☐	☒	Eukaryotic cell lines
☒	☐	Palaeontology and archaeology
☐	☒	Animals and other organisms
☐	☒	Clinical data
☒	☐	Dual use research of concern
☒	☐	Plants

Methods

n/a	Involved in the study	
☒	☐	ChIP-seq
☐	☒	Flow cytometry
☒	☐	MRI-based neuroimaging

Eukaryotic cell lines

Policy information about cell lines and Sex and Gender in Research

Cell line source(s)

All cell lines generated in this study were from mice of both male and female sex. MCA205, HeLa and RMA-S cell lines were obtained from the Francis Crick Institute Cell Services Science Technology platform. MuTu DC cells were obtained from Hans Acha-Orbea.

Authentication

The primary fibrosarcoma cell lines generated have been further characterised using the multiplex polymerase chain reaction (PCR) assay designed to profile mouse cell lines using primers targeting 18 mouse loci with highly polymorphic short tandem repeats (STRs), as previously described and validated by the Consortium for Mouse Cell Line Authentication (Almeida et al. 2019). This STR profiling was performed as part of the quality control process in curating a reference library for all the banked original primary cell lines, by the Cell Services Science Technology Platform at the Francis Crick Institute.

Mycoplasma contamination

All cell lines, including all the primary fibrosarcoma cell lines generated have been independently screened negative for mycoplasma contamination by the Cell Services Science Technology Platform at the Francis Crick Institute. In general, cells were cultured in antibiotic-free RPMI 1640 media for at least 2 days, before the submission of 5 to 10mL of confluent supernatant or the flask of live cells for simultaneous fluorescence staining and agar culture.

Commonly misidentified lines
(See ICLAC register)

n/a

Animals and other research organisms

Policy information about studies involving animals; ARRIVE guidelines recommended for reporting animal research, and Sex and Gender in Research

Laboratory animals

RAG1 KO (Rag1-/-), BATF3 KO (Batf3-/-), DNGR-1 KO (Clec9agfp/gfp or Clec9acre/cre), and WT (wild-type) mice on a C57BL/6 background were bred and maintained in specific-pathogen free conditions in the Biological Research Facility at The Francis Crick Institute. Experiments were commenced when mice were between 6 to 14 weeks old.

Wild animals

The study did not involve wild animals.

Reporting on sex

In all experiments, both male and female mice were used, unless otherwise indicated. In all loss-of-function experiments comparing DNGR-1 KO to WT mice, they were co-housed with sex- and age- matched WT controls for at least 3 weeks to eliminate any microbiota-dependent effects.

Field-collected samples

The study did not involve samples collected from the field.

Ethics oversight

All animal experiments were performed upon prospective approval of a study plan by the Biological Research Facility at the Francis Crick Institute, and strictly adhered to the Animals (Scientific Procedures) Act 1986.

Note that full information on the approval of the study protocol must also be provided in the manuscript.

Clinical data

Policy information about clinical studies
All manuscripts should comply with the ICMJE guidelines for publication of clinical research and a completed CONSORT checklist must be included with all submissions.

Clinical trial registration

For the bioinformatic analysis of human cancer datasets, the association between CLEC9A and survival outcomes in patients with cancer were analysed using publicly available data downloaded from the cBioPortal for Cancer Genomics platform [http://cbioportal.org/], with source data from The Cancer Genome Atlas (TCGA) Pan-cancer Atlas.

Study protocol

n/a

Data collection

n/a

Outcomes

The focus of our analyses were on survival outcomes, including progression-free survival and overall survival, provided by TCGA.

Plants

Seed stocks

n/a

Novel plant genotypes

n/a

Authentication

n/a

Flow Cytometry

Plots

Confirm that:

- [ ] The axis labels state the marker and fluorochrome used (e.g. CD4-FITC).
- [ ] The axis scales are clearly visible. Include numbers along axes only for bottom left plot of group (a 'group' is an analysis of identical markers).
- [ ] All plots are contour plots with outliers or pseudocolor plots.
- [ ] A numerical value for number of cells or percentage (with statistics) is provided.

Methodology

Sample preparation

Peptide pulsed RMA-S cells or regressor tumour cells were harvested in PBS/EDTA, washed and stained with appropriate anti-MHC-I mabs. Splenocyte suspensions were prepared from spleens of immunised mice, red blood cell lysed and stained with H-2Kb/SIINFEKL pentamer reagent, followed by anti-CD8α, anti-CD44 and anti-CD19 antibodies.

Instrument

Stained cells were acquired on a LSRFortessa (BD Biosciences).

Software

Data were analysed using FlowJo software (Treestar).

Cell population abundance

This study did not involve cell sorting. Cells analysed were either cell lines (RMA-S or regressor tumour lines) or total splenocytes. For each sample of RMA-S, regressor tumour or CD8+CD19- splenocytes at least 10000 events were recorded.

Gating strategy

CD8 T cells were first gated on scatter (FSC/SSC), followed by CD8/CD19. Gated CD8+CD19- cells were then plotted for CD44 and H-2Kb/OVA peptide pentamer and the percentage of OVA-specific CD8 T cells was determined from gating on H-2Kb/OVA peptide pentamer+CD44+cells.

- [ ] Tick this box to confirm that a figure exemplifying the gating strategy is provided in the Supplementary Information.
