# Supplementary figures and images for: Cross-presentation of dead cell-associated antigens shapes the neoantigenic landscape of tumor immunity
Source: Nat Immunol. 2026 Jan 2;27(1):72–81. doi: 10.1038/s41590-025-02354-w (PMC12764433; doi:10.1038/s41590-025-02354-w)

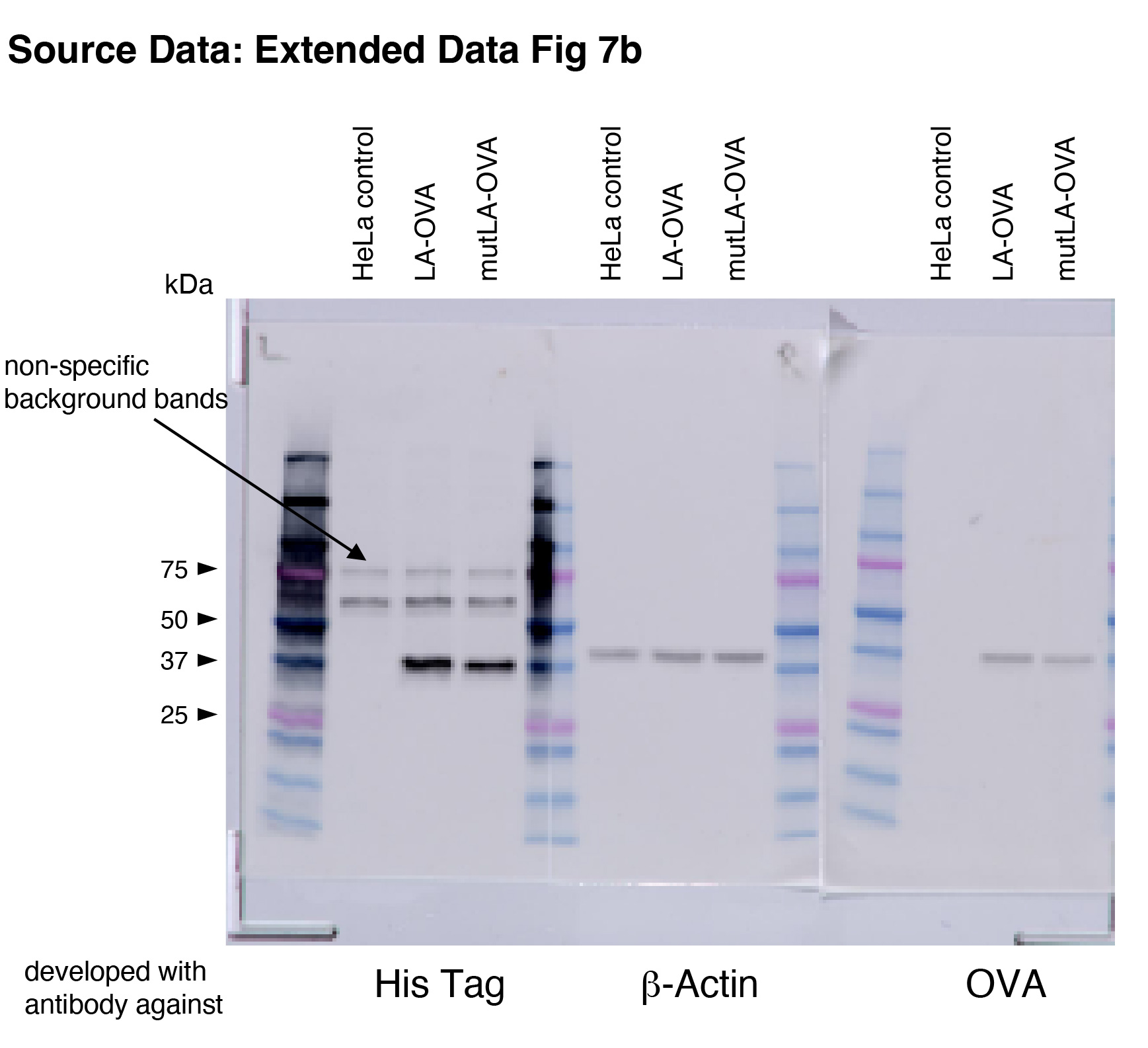

Supplement: Supplementary file 12 — Unprocessed western blots. [file 41590_2025_2354_MOESM12_ESM.jpg]
